# Risk of dementia and mild cognitive impairment in older adults with a criminal background: a population-based register study in Sweden

**DOI:** 10.1038/s41598-023-28962-w

**Published:** 2023-02-02

**Authors:** Carmen Solares, Miguel Garcia-Argibay, Zheng Chang, Maja Dobrosavljevic, Henrik Larsson, Henrik Andershed

**Affiliations:** 1 School of Behavioural, Social and Legal Sciences, Örebro University, Fakultetsgatan 1, 701 82 Örebro, Sweden; 2 School of Medical Sciences, Örebro University, Södra Grev Rosengatan 30, 703 62 Örebro, Sweden; 3 Department of Medical Epidemiology and Biostatistics, Karolinska Institutet, Stockholm, Sweden

**Keywords:** Psychology, Epidemiology, Risk factors, Outcomes research

## Abstract

Criminal behaviour has previously been associated with an increased risk for several mental health problems, but little is known about the association between criminal behaviour and dementia. We aimed to examine how the criminal background (type of crime, number of convictions, length of the sentence) is associated with dementia and mild cognitive impairment (MCI), and how mental and physical health disorders and educational attainment influenced these associations. A nationwide cohort of 3,617,028 individuals born between 1932 and 1962 were linked with criminal and medical records using Swedish national registers. We used Cox regression models to examine the associations. Increased risks for dementia (Hazard ratios (HRs) 1.54, 95% confidence interval (CI) 1.50–1.57) and MCI (1.55, 1.50–1.61) were found in individuals with criminal background, particularly among those who committed violent or several crimes, or with long sentences. After full adjustment of covariates, the associations attenuated but remained statistically significant for dementia (1.25, 1.22–1.28) and MCI (1.27, 1.22–1.32). The attenuation was mostly explained by mental health problems -depression, anxiety, schizophrenia spectrum disorders, substance use disorder (SUD), and bipolar disorder- (dementia: 1.34, 1.31–1.37; MCI: 1.35, 1.30–1.40). SUD contributed the most to attenuate the associations. Our results may provide important insights to health and penal systems by showing the importance of considering the severity of the criminal background and life-course mental health when assessing the risk of neurodegenerative disorders.

## Introduction

Dementia represents a major cause of disability among older adults worldwide^1^, but the risk of this disorder in older offenders and prisoners is still very understudied. Older adults with criminal background constitute a vulnerable and fast-growing population^2,3^. In Sweden, 15% of the offenders admitted to prisons in 2020 were older than 50 years^4^. In the United Kingdom the older prison population has increased by 243%, from 1511 to 5176 over the last two decades^5^. Despite the rising number of older offenders in the penal system, there are few studies on neurodegenerative disorders such as mild cognitive imparement (MCI) and dementia in this population including comparison groups. The prevalence of dementia and MCI in offenders is still unclear^6^. Although a recent meta-analysis^7^ showed a pooled prevalence of dementia of 6.9%, the heterogeneity between the included studies was high, which highlights the need of further research on this topic^8^. A better understanding of the risk of dementia and MCI among individuals with criminal background and of the potential role of health, psychosocial and criminal life-course factors may have implications for screening and intervention in forensic psychiatric services, prison settings and beyond.

Dementia refers to a group of disorders including Alzheimer´s disease and vascular dementia, which are usually the result of neurodegenerative processes and, which are characterized by impairment in cognition, behaviour, and daily functionality^1^. MCI is an early stage of a cognitive impairment where daily activities and functionality are preserved^9^. Previous research has showed a variety of life-course risk and protective factors (such as unhealthy habits, cardiovascular problems, depression, and education level) which can boost or delay the onset of certain types of dementia^10–14^. Interestingly, along their lifespan, offenders have been shown to be at increased risk for several physical and mental health problems such as hypertension, obesity, depression, substance use disorders (SUD), and traumatic brain injury, as well as a variety of psychosocial circumstances such as low educational level, low socio-economical level, loneliness, and poor physical activity^15–17^ which all have been described as risk factors for dementia^13,14^. Thus, it is possible that some of these factors or their combination put older adults with a criminal background at risk for the onset of dementia and MCI^6^.

The severity of the individual criminal background may also affect health and aging. For instance, individuals who recurrently committed crimes since adolescence, usually defined as persistent offenders, have a higher risk for adverse health outcomes than individuals with a more short-lived criminal career^18^. Persistent offenders tend to present antisocial behaviours from early on in life^19^, and they stand for most of the severe and serious crimes committed^20^. Thus, it may be of special interest for the penal and psychiatric systems where these individuals are usually referred, to know more about the aging and the risk of specific age-related disorders in these individuals. Unfortunately, previous studies have not investigated how different aspects of the criminal background (e.g., type of crime, number of offenses, sentence length) relate to dementia and MCI.

In the present study we explored if older adults with a criminal background have a higher risk for developing dementia and MCI in comparison to older adults without a criminal background. We also studied whether the risk for dementia and MCI varies depending on the severity of the criminal background. Specifically, we investigated whether older adults who committed several crimes, violent crimes or who had long sentences, present a higher risk for dementia and MCI. Finally, we studied how various mental and physical life-course disorders, and educational level could explain the potential higher risk for dementia and MCI among older adults with a criminal background.

## Results

The study cohort contained 3,617,028 individuals born between 1932 and 1962. We identified 792,230 (21.90%) people with criminal convictions, 617,139 (77.90%) were male. Table 1 presents descriptive statistics of individuals with and without criminal convictions. For descriptive characteristics of the other three measures of crime (i.e., type of crime, number of convictions, length of sentence) see supplementary material, Appendix C.

**Table 1:** Descriptive characteristics of the cohort according to criminal convictions.

Individuals study cohort	Overall N (%)	Criminal convictions^†^	
		No convictions N (%)	Convictions N (%)
Total	3,617,028	2,824,798 (78.10)	792,230 (21.90)
Male	1,847,582 (51.08)	1,230,443 (43.56)	617,139 (77.90)
Female	1,769,446 (48.92)	1,594,355 (56.44)	175,091 (22.10)
Dementia cases	56,590 (1.56)	45,400 (1.61)	11,190 (1.41)
Mean Age diagnosis (95% CI)	70.43 (70.38–70.49)	70.77 (70.70–70.83)	69.08 (68.95–69.21)
IRR 10,000 person years	10.81 (10.72–10.90)	10.7 (10.6–10.8)	11.24 (11.04–11.45)
Male, N (%)	27,978 (49.44)	19,479 (42.90)	8500 (75.96)
Mean Age diagnosis (95% CI)	70.13 (70.05–70.21)	70.62 (70.53–70.72)	69 (68.85–69.16)
IRR 10,000 person years	10.63 (10.50–10.75)	10.45 (10.31–10.60)	11.04 (10.81–11.28)
Female, N (%)	28,612 (50.56)	25,922 (57.10)	2690 (24.04)
Mean Age diagnosis (95% CI)	70.73 (70.65–70.81)	70.87 (70.79–70.96)	69.33 (69.05–69.61)
IRR 10,000 person years	11.87 (10.87–11.13)	10.91 (10.78–11.05)	11.94 (11.50–12.40)
MCI cases^‡^	23.394 (0.65)	18,179 (0.64)	5215 (0.66)
Mean Age diagnosis (95% CI)	67.83 (67.74–67.93)	68.29 (68.18–68.40)	66.24 (66.03–66.45)
IRR 10,000 person years	4.46 (4.40–4.52)	4.28 (4.22–4.34)	5.23 (5.09–5.37)
Male, N (%)	11,793 (50.41)	7912 (43.52)	3881 (74.42)
Mean Age diagnosis (95% CI)	67.80 (67.66–67.93)	68.56 (68.41–68.72)	66.23 (65.99–66.47)
IRR 10,000 person years	4.44 (4.39–4.55)	4.24 (4.14–4.33)	5.03 (4.87–5.19)
Female, N (%)	11,601 (49.59)	10,267 (56.48)	1334 (25.58)
Mean Age diagnosis (95% CI)	67.87 (67.73–68.01)	68.08 (67.93–68.23)	66.28 (65.86–66.70)
IRR 10,000 person years	4.45 (4.37–4.53)	4.31 (4.23–4.39)	5.9 (5.6–6.23)

*MCI* Mild Cognitive Impairment, *IRR* Incidence Rate Ratio per 10.000 person-years.

^†^Criminal Convictions is a time-varying exposure.

^‡^For the MCI analyses the study cohort consisted of 3,616,338 individuals. There were 690 individuals delated from the main cohort due to an MCI diagnosis established before age 50 or because 0 days of follow up.

We identified 56,590 (1.56%) people with a diagnosis of dementia after age 50, among who 27,978 (49.44%) were male. There were 23,394 (0.65%) people with a diagnosis of MCI after age 50, 11,793 (50.41%) were males. The mean follow-up time was 15.84 years for dementia and 14.49 years for MCI, with a maximum follow-up time of 31.95 years for both outcomes. Mean age at diagnosis for dementia and MCI was significantly lower in individuals with criminal convictions (mean age at diagnosis: for dementia = 69.08; for MCI = 66.24) compare to those without (mean age at diagnosis: for dementia = 70.77; for MCI = 68.29) (Table 1) (Wilcoxon Two-Sample Test: for dementia, Z = − 23.17, *P* < 0.0001; for MCI, Z = − 17.02, *P* < 0.0001). Educational attainment and all the health disorders showed a significant association for individuals with criminal convictions (adjusted for sex and birth year, Table 2).

**Table 2:** Descriptive characteristics of the cohort with regards to the mental and physical health disorders and educational attainment, and the associations with the criminal background (criminal convictions), presented as odds ratios (OR) with 95% confidence intervals (CI), adjusted for sex and birth year.

Health and psychosocial variables	Individuals without criminal convictions N = 2,824,798(%)	Individuals with criminal convictions N = 792,230 (%)	Total N = 3,617,028 (%)	OR (95% CI) adjusted for sex and birth year
Highest education (years)^†^	2,620,263	767,388	3,387,651	–
< 9^‡^	739,977 (26.20)	248,790 (31.40)	988,767 (27.34)	Ref.
Between 9 and 11	1,108,897 (39.26)	362,525 (45,76)	1,471,422 (40.68)	0.86 (0.85–0.87)
≥ 12	771,389 (27.31)	156,073 (19.70)	927,462 (25.64)	0.51 (0.50–0.52)
Depression	130,600 (4.62)	65,114 (8.22)	195,714 (5.41)	2.33 (2.30–2.35)
Anxiety	55,085 (1.95)	25,994 (3.28)	81,079 (2.24)	2.26 (2.23–2.30)
Substance use disorder	85,089 (3.01)	124,643 (15.73)	209,732 (5.8)	5.89 (5.84–5.95)
Schizophrenia spectrum disorders	39,321 (1.39)	23,352 (2.95)	62,673 (1.73)	2.49 (2.45–2.54)
Bipolar disorder	26,344 (0.9)	13,099 (1.65)	38.443 (1.06)	2.32 (2.27, 2.37)
Head injuries	103,680 (3.67)	75,813 (9.57)	179,493 (4.96)	2.59 (2.57–2.62)
Hypertension	569,195 (20.15)	168,385 (21.25)	737,580 (20.39)	1.22 (1.21–1.3)
Type 2 diabetes	184,588 (6.53)	71,377 (9.01)	255,965 (7.08)	1.46 (1.44–1.47)
Obesity	63,922 (2.26)	26,725 (3.37)	90,647 (2.51)	1.82 (1.80–1.85)
Hyperlipidaemia	199,014 (7.05)	66,016 (8.33)	265,030 (7.33)	1.20 (1.19–1.21)
Cerebrovascular diseases	150,698 (5.33)	54,488 (6.88)	205,186 (5.67)	1.46 (1.45–1.48)

^†^Highest education: there are 229,377 missing values: 204,535 missing values for Individuals without criminal convictions, 24,842 missing values for individuals with criminal convictions.

^‡^References category.

We found an increased risk of having dementia diagnosis (HR 1.54, 95% CI 1.50–1.57) and MCI (HR 1.55, 95% CI 1.52–1.59) in individuals with criminal convictions compared to individuals without criminal convictions, adjusting for sex and birth year (Tables 3, 4). After full adjustment, we observed that the risk for both outcomes was attenuated but still significantly higher for individuals with criminal convictions in comparison to individuals without (Dementia: HR 1.25, 95% CI 1.22–1.28; MCI: HR 1.27, 95% CI 1.22–1.32). After separate adjustments of the analyses for the different sets of covariates: educational attainment (model 3), mental disorders (model 4) and physical disorders (model 5), we observed that mental disorders reduced the risk for dementia and MCI the most (Dementia: HR 1.34, 95% CI 1.31–1.37; MCI: HR 1.35, 95% CI 1.30–1.40). Separate follow-up analyses adjusting for each mental disorder, in addition to sex and year of birth, showed that substance use disorders had the strongest influence on the association between the exposure and both outcomes (see Tables 3, 4). To further illustrate the impact of SUD on the associations we performed post-hoc analysis comparing individuals with criminal convictions with and without a SUD diagnosis. After full adjustment for all covariates, we found that individuals with criminal convictions and SUD had a higher risk of dementia and MCI compared with individuals with criminal convictions but without SUD (Dementia: HR 1.46, 95% CI 1.41–1.52; MCI: HR 1.22, 95% CI 1.12–1.33).

**Table 3:** Association between the four measures for the criminal background and dementia as hazard ratios (HR) with 95% confidence intervals (CI).

Measures criminal background	N	Model 1	Model 2	Model 3	Model 4	Model 5	Model 6	Model 7	Model 8	Model 9	Model 10
*Criminal conviction*											
No-conviction	2,824,798	Ref.	Ref.	Ref.	Ref.	Ref.	Ref.	Ref.	Ref.	Ref.	Ref.
Conviction	792,230	1.54 (1.50–1.57)	1.25 (1.22–1.28)	1.48 (1.44–1.51)	1.34 (1.31–1.37)	1.47 (1.43–1.50)	1.46 (1.42–1.49)	1.51 (1.47- 1.55)	1.38 (1.35–1.42)	1.50 (1.47–1.53)	1.51 (1.48–1.55)
*Type of crime*^†‡^											
No crime	2,824,798	Ref.	Ref.	Ref.	Ref.	Ref.	Ref.	Ref.	Ref.	Ref.	Ref.
Non-violent	737,415	1.45 (1.42–1.49)	1.22 (1.19–1.25)	1.40 (1.37–1.44)	1.30 (1.27–1.33)	1.35 (1.32–1.39)	1.39 (1.36–1.43)	1.43 (1.40–1.47)	1.33 (1.30–1.36)	1.44 (1.40–1.47)	1.44 (1.40–1.47)
Violent	118,155	1.97 (1.86–2.09)	1.34 (1.27–1.43)	1.91 (1.80–2.02)	1.43 (1.35–1.52)	1.77 (1.67–1.87)	1.74 (1.64–1.80)	1.89 (1.70–2.00)	1.58 (1.48–1.67)	1.78 (1.68–1.79)	1.88 (1.78–2.00)
*Length of sentence (months)*^§^											
0	3,495,430	Ref.	Ref.	Ref.	Ref.	Ref.	Ref.	Ref.	Ref.	Ref.	Ref.
1–11	94,887	2.28 (2.15–2.42)	1.60 (1.50–1.70)	2.20 (2.07–2.33)	1.72 (1.61–1.83)	2.14 (2.02–2.27)	2.09 (1.97–2.22)	2.23 (2.10–2.36)	1.68 (1.58–1.79)	2.23 (2.11–2.37)	2.26 (2.13–2.40)
12–23	11,010	2.30 (1.89–2.80)	1.55 (1.27–1.90)	2.28 (1.87–2.78)	1.66 (1.36–2.03)	2.15 (1.76–2.61)	2.07 (1.69–2.52)	2.22 (1.82–2.70)	1.68 (1.38–2.05)	2.20 (1.81–2.68)	2.25 (1.84–2.74)
≥ 24	16,701	2.47 (2.08–2.93)	1.63 (1.38–1.94)	2.44 (2.05–2.89)	1.78 (1.50–2.11)	2.34 (1.97–2.77)	2.20 (1.85–2.61)	2.39 (2.02–2.83)	1.79 (1.51–2.12)	2.34 (1.98–2.78)	2.43 (2.05–2.88)
*Number of conviction*^§^											
0	2,824,248	Ref.	Ref.	Ref.	Ref.	Ref.	Ref.	Ref.	Ref.	Ref.	Ref.
1	454,983	1.62 (1.58–1.67)	1.44 (1.40–1.48)	1.57 (1.53–1.62)	1.52 (1.48–1.56)	1.55 (1.50–1.59)	1.57 (1.53–1.62)	1.61 (1.56–1.65)	1.55 (1.51–1.60)	1.61 (1.56–1.65)	1.61 (1.57–1.66)
2–3	210,019	2.02 (1.93–2.11)	1.58 (1.51–1.65)	1.93 (1.85–2.02)	1.71 (1.64–1.79)	1.84 (1.76–1.92)	1.88 (1.80–1.97)	1.97 (1.89–2.06)	1.79 (1.71–1.87)	1.97 (1.88–2.05)	1.97 (1.89–2.06)
4–9	95,871	2.61 (2.45–2.78)	1.75 (1.64–1.88)	2.49 (2.33–2.65)	1.94 (1.81–2.07)	2.25 (2.11–2.40)	2.32 (2.23–2.41)	2.51 (2.35–2.68)	2.05 (1.92–2.19)	2.48 (2.33–2.65)	2.52 (2.37–2.69)
10–19	21,370	3.48 (3.02–4.02)	1.97 (1.71–2.27)	3.30 (2.87–3.8)	2.21 (1.91–2.55)	2.85 (2.48–3.28)	2.97 (2.58–3.42)	3.29 (2.86–3.79)	2.36 (2.05–2.73)	3.16 (2.74–3.64)	3.29 (2.86–3.79)
≥ 20	10,537	4.94 (4.04–6.03)	2.51 (2.03–3.09)	4.66 (3.81–5.70)	2.99 (2.44–3.65)	3.72 (3.03–4.56)	4.24 (3.47–5.18)	4.68 (3.83–5.71)	3.02 (2.47–3.71)	4.39 (3.59–5.36)	4.75 (3.89–5.81)

Model 1: HR (95% CI) adjusted for sex and birth year. Model 2: Full Adjustment for all covariates. Model 3: HR (95% CI) adjusted for sex and birth year and educational attainment. Model 4: HR (95% CI) adjusted for sex and birth year and mental health disorders. Model 5: HR (95% CI) adjusted for sex and birth year and physical health disorders. Model 6: HR (95% CI) adjusted for sex and birth year and depression. Model 7: HR (95% CI) adjusted for sex and birth year and anxiety. Model 8: HR (95% CI) adjusted for sex and birth year and substance use disorder. Model 9: HR (95% CI) adjusted for sex and birth year and schizophrenia spectrum disorders. Model 10: HR (95% CI) adjusted for sex and birth year and bipolar disorder.

*N* Number of individuals.

^†^Type of crime is a time-varying exposure.

^‡^Type of crime: individuals with convictions can contribute to both non-violent and violent types of crime.

^§^Number of convictions and Length of the sentence are time-fixed exposures showing the total number of convictions or sentence months in the individual´s lifetime.

**Table 4:** Association between the four measures for the criminal background and mild cognitive impairment (MCI) as hazard ratios (HR) with 95% confidence intervals (CI).

Measures criminal background	N	Model 1	Model 2	Model 3	Model 4	Model 5	Model 6	Model 7	Model 8	Model 9	Model 10
*Criminal conviction*											
No-conviction	2,824,798	Ref.	Ref.	Ref.	Ref.	Ref.	Ref.	Ref.	Ref.	Ref.	Ref.
Conviction	792,230	1.55 (1.50–1.61)	1.27 (1.22–1.32)	1.51 (1.46–1.57)	1.35 (1.30–1.40)	1.47 (1.42–1.53)	1.44 (1.39–1.50)	1.52 (1.47–1.57)	1.40 (1.35–1.45)	1.52 (1.47–1.58)	1.52 (1.47–1.58)
*Type of crime*^†‡^											
No crime	2,824,798	Ref.	Ref.	Ref.	Ref.	Ref.	Ref.	Ref.	Ref.	Ref.	Ref.
Non-violent	737,415	1.50 (1.45–1.56)	1.26 (1.22–1.31)	1.46 (1.41–1.52)	1.34 (1.29–1.39)	1.39 (1.34–1.44)	1.42 (1.37–1.47)	1.48 (1.42–1.53)	1.38 (1.33–1.43)	1.49 (1.43–1.54)	1.48 (1.43–1.54)
Violent	118,155	1.54 (1.41–1.69)	1.11 (1.02–1.22)	1.56 (1.43–1.71)	1.13 (1.03–1.24)	1.37 (1.25–1.50)	1.33 (1.21–1.45)	1.47 (1.34–1.60)	1.24 (1.13–1.36)	1.43 (1.30–1.56)	1.46 (1.34–1.60)
*Length of sentence (months)*^§^											
0	3,495,430	Ref.	Ref.	Ref.	Ref.	Ref.	Ref.	Ref.	Ref.	Ref.	Ref.
1–11	94,887	1.76 (1.60–1.93)	1.27 (1.15–1.40)	1.76 (1.61–1.94)	1.33 (1.20–1.46)	1.63 (1.49–1.80)	1.57 (1.42–1.72)	1.70 (1.50–1.87)	1.31 (1.18–1.44)	1.73 (1.57–1.90)	1.73 (1.57–1.90)
12–23	11,010	2.06 (1.56–2.72)	1.43 (1.08–1.89)	2.12 (1.61–2.81)	1.47 (1.11–1.95)	1.90 (1.44–2.51)	1.80 (1.36–2.38)	1.98 (1.49–2.61)	1.49 (1.12–1.97)	1.98 (1.50–2.62)	1.99 (1.51–2.63)
≥ 24	16,701	2.40 (1.88–3.06)	1.64 (1.28–2.09)	2.48 (1.95–3.16)	1.70 (1.33–2.17)	2.25 (1.77–2.86)	2.06 (1.61–2.62)	2.30 (1.81–2.93)	1.69 (1.32–2.16)	2.29 (1.79–2.91)	2.36 (1.85–3.00)
*Number of conviction*^§^											
0	2,824,248	Ref.	Ref.	Ref.	Ref.	Ref.	Ref.	Ref.	Ref.	Ref.	Ref.
1	454,983	1.69 (1.62–1.77)	1.49 (1.43–1.56)	1.64 (1.57–1.72)	1.57 (1.51–1.65)	1.60 (1.53–1.67)	1.62 (1.55–1.69)	1.67 (1.60–1.75)	1.62 (1.55–1.70)	1.68 (1.60–1.75)	1.67 (1.60–1.75)
2–3	210,019	2.03 (1.90–2.16)	1.61 (1.51–1.72)	1.98 (1.86–2.11)	1.72 (1.61–1.83)	1.83 (1.72–1.95)	1.85 (1.73–1.97)	1.97 (1.85–2.10)	1.81 (1.69–1.93)	1.98 (1.86–2.11)	1.98 (1.86–2.11)
4–9	95,871	2.32 (2.11–2.55)	1.62 (1.47–1.79)	2.31 (2.10–2.53)	1.72 (1.56–1.90)	1.98 (1.80–2.17)	1.99 (1.81–2.19)	2.21 (2.01–2.43)	1.85 (1.67–2.04)	2.23 (2.03–2.45)	2.23 (2.03–2.45)
10–19	21,370	2.87 (2.34–3.51)	1.74 (1.41–2.15)	2.89 (2.41–4.39)	1.86 (1.51–2.29)	2.32 (1.89–2.84)	2.34 (1.91–2.87)	2.68 (2.19–3.29)	1.97 (1.60–2.43)	2.66 (2.17–3.26)	2.69 (2.19–3.30)
≥ 20	10,537	3.17 (2.35–4.27)	1.81 (1.33–2.45)	3.26 (2.73–3.88)	1.93 (1.43–2.62)	2.40 (1.78–3.24)	2.59 (1.92–3.50)	2.95 (2.19–3.98)	1.94 (1.43–2.63)	2.87 (2.13–3.87)	3.00 (2.22–4.03)

Model 1: HR (95% CI) adjusted for sex and birth year. Model 2: Full Adjustment for all covariates. Model 3: HR (95% CI) adjusted for sex and birth year and educational attainment. Model 4: HR (95% CI) adjusted for sex and birth year and mental health disorders. Model 5: HR (95% CI) adjusted for sex and birth year and physical health disorders. Model 6: HR (95% CI) adjusted for sex and birth year and depression. Model 7: HR (95% CI) adjusted for sex and birth year and anxiety. Model 8: HR (95% CI) adjusted for sex and birth year and substance use disorder. Model 9: HR (95% CI) adjusted for sex and birth year and schizophrenia spectrum disorders. Model 10: HR (95% CI) adjusted for sex and birth year and bipolar disorder.

*N* Number of individuals.

^†^Type of crime is a time-varying exposure.

^‡^Type of crime: Individuals with convictions can contribute to both non-violent and violent types of crime.

^§^Number of convictions and Length of the sentence are time-fixed exposures showing the total number of convictions or sentence months in the individual´s lifetime.

These patterns of results for the partially and fully adjusted analyses were similar to the results for the association between the other three measures of crime (i.e., type of crime, number of convictions and length of the sentence) and the outcomes. Individuals who committed violent crimes, with several convictions and with long sentences, showed higher risk for dementia and MCI than individuals without criminal convictions, even after full adjustment (Violent crime: Dementia: HR 1.34, 95% CI 1.27–1.43; MCI: HR 1.11, 95% CI 1.02–1.22; Between 2 and 3 Convictions: Dementia: HR 1.58, 95% CI 1.51–1.65; MCI: HR 1.61, 95% CI 1.51–1.72; Between 4 and 9 Convictions: Dementia: HR 1.75, 95% CI 1.64–1.88; MCI: HR 1.62¸95% CI 1.47–1.79; Between 1 and 11 months of imprisonment: Dementia: HR 1.60, 95% CI 1.50–1.70; MCI: HR 1.27, 95% CI 1.15–1.40) (see Tables 3, 4 for a complete report of the analysis). In addition, mental disorders, and more specifically SUD, attenuated the most the risk for both outcomes in all the analyses.

### Sensitivity analyses

Sensitivity analyses yielded results consistent with the partially and fully adjusted analyses for both dementia and MCI outcomes, except for the highest categories of the variables; number of convictions (more than 20 convictions) and length of the sentence (more than 24 months). For both measures of crime (i.e., number of convictions, length of sentence) the HRs for the association between the highest category of each variable with dementia and MCI were no longer significant or decreased in comparison to less severe categories (supporting tables, see supplementary material, Appendix D).

## Discussion

In this large-scale and longitudinal register-based cohort study, we found that older adults with criminal background had a higher risk for both dementia and MCI in comparison to older adults without criminal background. Additionally, we found an increased risk for dementia for individuals with more severe criminal backgrounds, that is, individuals who committed violent crimes, had longer sentence periods or committed several crimes. The increased risk for dementia and MCI was influenced by a combination of negative health and psychosocial life-course factors, where mental health problems attenuated the most the associations, and with substance use disorders contributing the most to these attenuations. To our knowledge, this is the first time ever the association between the severity of the criminal background and dementia and MCI has been examined with adjustment for potential life-course risk factors.

Our finding of an increased risk of dementia and MCI in those older adults with criminal background is in line with recent studies showing a more marked cognitive impairment in older prisoners in comparison to older adults in the community^21–23^; even thought these studies are scarce and have small sample sizes. The strength of the association between criminal background and dementia are comparable to the relative risks previously reported for known dementia risk factors (OR range from 1.46 to 1.96), such as depression, anxiety^14^, midlife hypertension, diabetes mellitus or low education^13^. Increased clinical awareness of neurodegenerative disorders in people with criminal background is probably warranted. Moreover, considering previous evidence showing associations between violent behaviour and mental health problems^16,24,25^, and indicating high psychiatric comorbidity among offenders^17^, our results may be especially relevant for outpatient mental health services to which offenders may be referred along their life-course or after being released. Nevertheless, further research is needed to identify the underlying mechanisms of these associations and to design more adjusted clinical assessments and intervention protocols for individuals with a criminal background.

We found that the severity of the criminal background is associated with a higher risk of dementia and MCI in older adults. In other words, the risk increased for those individuals who committed violent crimes, several crimes or had longer sentences. Interestingly, some of the risk factors for dementia have also been described as risk factors for violent behaviour. For instance, mental health problems such as depression or SUD, or family-related factors such as low socio-economic status or low educational attainment^26,27^, seem to increase the risk of dementia but they have been also associated with a higher risk of perpetrating violent crimes^16,25,28,29^. In addition, previous research^30^ has suggested that inside prison older adults may be exposed to social deprivation, physical and mental inactivity or poor nutrition that may potentially act as risk factors for the onset of dementia and MCI and therefore, to increase the risk for these disorders among older prisoners. Taken together, our results suggest that criminal background, particularly crime severity, may be considered when assessing the risk for neurodegenerative disorders in offenders and when designing preventive and interventive health programs for older offenders inside prison or when re-entering their communities.

We observed that mental and physical disorders, especially SUD, substantially attenuated the risk for dementia and MCI in older adults with criminal background. Our findings are consistent with studies from community-dwelling older adults demonstrating the impact of several life-course risk factors on dementia development, including chronic alcohol consumption, depression, severe psychiatric disorders, cardiovascular diseases, obesity, lack of exercise, smoking, metabolic disorders, traumatic brain injuries and certain psychosocial factors such as low educational level^10–13,31,32^. Previous research has suggested that dementia may be reduced through better access to education and reducing the prevalence of vascular life-course risk factors^9,10,26,33^. However, in our study, mental disorders decreased to a major extent the risk of dementia and MCI in comparison to physical disorders and educational attainment, and SUD was the mental disorder that contributed the most to the attenuation of the associations. There is an extensive body of evidence showing cognitive decline in drug and alcohol use disorders^34^, and the Fifth Edition of the Diagnostic and Statistical Manual of Mental Disorders (DSM-V) includes specific diagnostic criteria for Substance-induced mild and major neurocognitive disorder. Impairment in different cognitive skills like executive function, memory and attention has been also reported in patients with depression^35^. For instance, “Pseudodementia” is a clinical phenomenon which describes a psychiatric condition, usually depression, that affects cognition and gives the appearance of a neurocognitive disorder^36^. Thus, it is important to consider that a misdiagnosis of dementia may also influence the observed associations for the studied population. Therefore, our results showed that the increased risk for dementia in older adults with criminal background may be mainly influenced by the combination of negative health (especially mental health) and psychosocial life-course factors. Nevertheless, due to the high prevalence of psychiatric comorbidities in this population, special attention may be given to differential diagnosis between dementia and other psychiatric disorders that run with similar clinical cognitive impairments. Furthermore, it may be beneficial for the mental health and penal services where these individuals may be remitted to be aware of the increased risk for MCI and dementia in this population, and to include in their assessment routines screening measures for mild and major neurocognitive disorders as preventive strategy.

This study has several limitations. First, there was information about other life-course risk factors that we could not cover since data were not available in the registers. Some examples are feelings of loneliness, physical exercise, smoking, daily functionality, or premorbid cognitive abilities. Furthermore, early emerging neurocognitive problems and genetic variables may also need to be considered as potential factors influencing the observed associations. For instance, prefrontal deficit dysfunctions have been related with neurodevelopmental and neurodegenerative disorders, traumatic brain injury and severe psychiatric disorders, but also with criminal behaviour^37,38^. Because we lacked information about neurocognitive or other genetic variables, future research may explore whether prefrontal structural and functional impairments or other biological predispositions can potentially account for the increased risk of dementia in older adults with criminal background. Second, due to the nature of the Swedish national registers and our study design, we were only able to follow most of the individuals until they were approximately 70 years old, whereas the onset of dementia usually peaks around 80 years of age^39^. Thus, our findings may primarily reflect earlier onset dementia. Replication studies are therefore needed in samples based on individuals followed across the full at-risk period for dementia. Third, the rates of criminal convictions were higher in our study compared to previous research^40^. Our study was done in one country where information on health disorders and criminal convictions was extracted from Swedish registry data recorded over several decades. The use of the national Swedish Crime Register—which in principle contains complete nationwide data on all convictions since 1973—to extract information about criminal convictions, is one potential explanation to observed differences. Further, replication studies are needed to explore if our findings generalize to countries and settings with lower rates of criminal convictions. Fourth, the results from our sensitivity analysis, where we restricted the time to be exposed to a nine years-time-window before the individuals turn 50 years old, revealed that the associations between the highest category of number of convictions and length of the sentence with dementia and MCI were less strong compared to the results of the main analysis. One potential explanation to the somewhat different results in the sensitivity analysis compared to the main analysis relates to the crime-age curve^41^. The age-crime curve shows how individuals tend to commit most of the crimes in the adolescent and early adulthood periods with a marked drop in criminal behaviour in later life periods. Thus, the time-window in the sensitivity analysis may be too restricted to capture the accumulation of a high number of convictions or sentence months in the studied population, which decreased the sample size in the most severe categories, which in turn influenced the statistical power of the analysis. Fifth, we lacked statistical power to explore the associations between different aspects of the criminal background with specific types of dementia (e.g., vascular dementia, frontotemporal dementia). Sixth, although our study used a prospective design, we could not completely rule out that part of the observed associations was due to reverse causation. For example, aggressive behaviour is a common issue for dementia patients and their families, these behaviours are considered as Noncognitive Neuropsychiatric Symptoms (NPS) of dementia^42^, and they could manifest years before the individual receives a dementia diagnosis^43^. Finally, another potential limitation is the misclassification of both dementia and MCI cases. Many individuals with cognitive and psychiatric problems may not seek professional attention and thus never given a diagnosis. Moreover, it is possible that not all dementia and MCI cases among older prisoners are diagnosed due to the lack of special geriatric units, medical and follow-up assessments for older offenders in the prison system.

## Conclusions

Older adults with criminal background, and specially those who committed violent crimes, several crimes or who had long sentences, are at higher risk for dementia and MCI. The risk for both outcomes is influenced by a combination of health and psychosocial life-course factors, with mental health problems and especially substance use disorders being major risk factors. Our findings provide initial but important insights into clinical (e.g., outpatient psychiatric services) and public systems (e.g., forensic psychiatric and prison settings) by showing the importance of considering crime severity and life-course mental health when assessing the risk of neurodegenerative disorders. These results may contribute to the development of adequate preventive and interventive programs to reduce and treat the impact of these disorders when offenders and prisoners age. Future research is needed to understand which other genetic, familial, and life-course aspects may affect the associations.

## Methods

### Data sources

We used data from the linkage of Swedish population-based registers via the unique personal identification numbers that are assigned to all individuals in Sweden^44^. The Total population register (TPR) includes demographic information on all Swedish inhabitants since 1968^44^. The National Patient Register (NPR) contains information on all in-patient diagnosis from 1987 and on out-patient visits from 2001^45^. The Cause of Death Register (CDR) contains information of all deaths since 1952^46^. Diagnoses in NPR and CDR are coded using the International Classification of Diseases (ICD) versions 7/8/9/10. The Prescribed drug register (PDR) includes all prescribed drugs dispensed in Sweden since July 2005^47^, using the Anatomical Therapeutic Classification (ATC) system. Longitudinal integration database for health insurance and labour market studies register (LISA) includes data on educational attainment for all individuals aged ≥ 16 since 1990^48^. National crime register (NCR) contains records of all criminal convictions in Swedish law courts since 1973^24^.

### Study population

We selected a cohort of all people older than 50 years old who were alive and living in Sweden at age 50 between the years 1982 and 2013. We excluded from the cohort and further analyses all individuals who had a dementia diagnosis, migrated, or died before the start of follow-up.

### Measures of crime

Four different measures of crime were constructed to cover different aspects of the severity of the criminal background: (1) Criminal conviction: to be convicted for any type of crime; (2) Type of crime: to have committed a violent or non-violent crime. Based on previous literature violent crime was defined as murder, homicide, assault, robbery, arson, any sexual offense (rape, sexual coercion, child molestation, indecent exposure, or sexual harassment), illegal threats, or intimidation^28,49^; (3) Length of the sentence: Total number of prison months accumulated along the lifetime of an individual. The variable was divided into no imprisonment, 1–11 months, 12- 23 months, and ≥ 24 months; (4) Number of convictions: Total number of convictions along the lifetime of an individual. This variable was divided into No convictions, one conviction, two–three convictions, four-nine convictions, 10–19 convictions and ≥ 20 convictions.

### Measures of dementia and MCI

Dementia and MCI cases were defined as a registered diagnosis for Alzheimer’s disease, vascular dementia, and other dementias (see supplementary material, Appendix A for a complete report of the types of dementias included in the study; for validity of dementia diagnosis see^50^). We included a diagnosis of dementia or MCI recorded at age 50 or older. Diagnoses were collected from the NPR and the CDR with diagnostic codes according to the ICD-8/9/10, or from a medication prescription for Alzheimer’s disease according to the ATC codes from the PDR. Cases of MCI were identified from the NPR in accordance with the ICD-10. For the ICD and ATC codes^51^ of both dementia and MCI diagnosis, see supplementary material, Appendix A.

### Covariates

Based on previous literature we selected as covariates variables that have been described as risk factors for dementia^10–14^ and are common health and psychosocial problems along the life-course of offenders^15–17^. Specifically, we selected from the NPR diagnosis of several physical disorders (i.e., type 2 diabetes, hypertension, hyperlipidemia, obesity, cerebrovascular disorders, traumatic brain injury) and mental disorders (i.e., depression, anxiety, SUD, schizophrenia spectrum disorders and bipolar disorders; see supplementary material, Appendix B for the ICD codes). In addition, we collected from LISA information about educational attainment: ≤ 9 years (compulsory education), 10–11 years (upper secondary), ≥ 12 years (post-secondary).

### Statistical analyses

In the main analyses, separate Cox proportional hazards models were performed for each of the four crime measures to explore the association between criminal background and dementia or MCI, with age as the underlying time scale in the analyses. Hazard ratios (HRs) were estimated with 95% confidence intervals (CIs). Individuals were followed since the day they turned 50 years old to date of diagnosis of dementia or MCI, emigration, death, or the end of follow-up in December 2013, which ever came first. Criminal conviction and type of crime were entered as time-varying exposures. Individuals with criminal convictions prior to age 50 were considered as exposed from start of follow-up, while individuals convicted for crime during the follow-up period contributed to the exposed group from the conviction date onwards. In addition, type of crime followed a hierarchical time-varying approach where an individual could enter the follow-up as unexposed to crime and then being exposed to non-violet or violent crime during the follow-up period. But also, an individual could enter the follow-up as exposed to non-violent crime and then being exposed to violent crime during the follow-up period. The exposures number of convictions and length of the sentence as well as the covariate educational attainment entered the analyses as time-fixed variables. All mental and physical health covariates were analyzed as time-varying covariates.

Covariates were entered in separate regression models. Model 1 adjusted for sex and year of birth and Model 2 fully adjusted for sex and year of birth and all the covariates. To understand differential influences of the covariates to the associations, we ran separate partially adjusted analyses for the different sets of covariates in addition to sex and year of birth: Model 3 adjusted for educational attainment; Model 4 adjusted for all mental disorders; Model 5 adjusted for all physical disorders. Finally, we performed further partially adjusted models for each specific disorders included in the set of covariates that had the strongest effect on the associations (Models 6–10).

We used Wilcoxon two-sample test to evaluate differences in age of dementia/MCI diagnosis between individuals with and without criminal background. In case that distribution of frequencies for the risk factors differed between groups with and without criminal background, we used logistic regression models to inspect associations between crime conviction and each specific risk factor included as covariates in the analysis, presented as odds ratios (OR) with 95% CIs and adjusted for sex and birth year.

Sensitivity analyses were performed for two of the exposures: number of convictions and length of sentence. Records in the NCR started in 1973 and the oldest individuals in our cohort turned 50 years old in 1982 meaning that they only had the possibility of being in the NCR for 9 years before they turn 50. For the sensitivity analyses we limited the time to be exposed to a time-window of 9 years before the individuals turn 50 years old. We decided on this time-window for two reasons: (1) to explore whether the results were influenced by the definition of these exposures (i.e., lifetime versus fixed-time period for being exposed) and (2) to allow all individuals in the cohort the same amount of time for being exposed.

All methods were performed in accordance with relevant guidelines and regulations. SAS version 9.4 (SAS Institute, Inc.) was used for data management and R version 4.1.0 for data analyses.

### Ethical approval

Ethical approval for this study was granted by the regional ethics review board in Stockholm, Sweden (approval numbers: Dnr.2013/862-31/5, Dnr. 2017/1985-32). Individual informed consent was waived due to the database nature of the study^44,52^.

## Supplementary Information

Supplementary Information.

## Data Availability

Restrictions apply to the availability of the data that supports the findings of this study. The Public Access to Information and Secrecy Act in Sweden prohibits individual level data to be publicly available. Researchers who are interested in replicating this study can apply for individual level data at Statistics Sweden: www.scb.se/en/services/guidance-for-researchersand-universities/.
