# Supplementary material for: Risk of dementia and mild cognitive impairment in older adults with a criminal background: a population-based register study in Sweden
Source: Sci Rep. 2023 Feb 2;13:1915. doi: 10.1038/s41598-023-28962-w (PMC9894846; doi:10.1038/s41598-023-28962-w)
Supplement: Supplementary file 1 — Supplementary Information. [file 41598_2023_28962_MOESM1_ESM.pdf]

# Risk of dementia and mild cognitive impairment in older adults with a criminal background: A population-based register study in Sweden.

Carmen Solares*, Miguel Garcia-Argibay, Zheng Chang, Maja Dobrosavljevic, Henrik Larsson, Henrik Andershed.

* Corresponding author: Carmen Solares, E-mail: carmen.solares-canal@oru.se

**Supplementary Material.**

Appendix A. OUTCOMES: Dementia and Mild Cognitive Impairment CODES.

Appendix B. COVARIATES: Codes for mental and physical health disorders and Educational Attainment.

Appendix C. DESCRIPTIVE TABLE: Descriptive characteristics of the cohort according to the following measures of crime: Type of crime, Number of convictions and Length of the sentences

Appendix D. SENSITIVITY ANALYSIS

## Appendix A. OUTCOMES: Dementia and Mild Cognitive Impairment CODES:

| Outcome | ICD-7† (1964-1968) | ICD-8† (1969-1986) | ICD-9† (1987-1996) | ICD-10† (1997-2013) | ATC‡ (2005-2014) |
|---|---|---|---|---|---|
| **Alzheimer (AD)** | 304-305 | 290 | 290A/B/X, 331A | F00, F03, G30 | N06DA02-N06DA04, N06DX01 |
| **Vascular dementia** | 306 | 293.0-293.1 | 290E | F01 | - |
| **Other dementia §** | - | - | 294B, 290W, 331B/C/X | F02, F02.1, F02.2, F02.3, F02.4, F02.8, F05.1, G31.1, G31.8, F02.8 | - |
| **Mild cognitive impairment (MCI)** | - | - | - | F06.7 | - |

*NOTE. † ICD Codes for MCI and Dementia diagnosis from the NPR and the CDR. ‡ ATC codes from PDR. § 294B= Dementia associated with other cerebral diseases. Persistent mental disorders due to conditions classified elsewhere; 290w = Dementias (including senile dementia, presenile, vascular dementia and other unspecified senile conditions); 331B = Pick's disease; 331C Senile degeneration of the brain of unspecified type; 331X Cerebral degeneration, unspecified; F02.1-f02-4: Dementia in Parkinson's disease, Dementia in Pick's disease; Dementia in Huntington's disease; Dementia in disease caused by human immunodeficiency virus [HIV]; Dementia in other specified diseases classified elsewhere; Delirium with underlying dementia; Other specified degenerative diseases of the nervous system; Senile degeneration of the brain not elsewhere classified.*

**APPENDIX B. COVARIATES: Codes for mental and physical health disorders:**

| Covariate | ICD-8† (1969-1986) | ICD-9† (1987-1996) | ICD-10† (1997-2013) |
|---|---|---|---|
| **Hypertension** | 400 – 404 | 401 – 405 | I10 – I13, I15 |
| **Type 2 diabetes mellitus** | NA | NA | E11 |
| **Obesity** | 277,99 | 278A, 278B | E65-E66 |
| **Head Injuries** | 800, 801, 803, 850-854 | 800, 801, 803, 850-854 | S020, S021, S027-S029, S060-S071 |
| **Hyperlipidaemia** | 279 | 272 | E78 |
| **Cerebrovascular disease** | 430-438 | 430-438 | I60-I69 |
| **Depression** | 296.0, 300.4 | 296B, 300E, 311 | F32, F33 |
| **Anxiety** | 300 (except 300.4) | 300 (except 300E) | F40, F41, F42, F44, F45, F48 |
| **Substance use disorder** | 303, 304 | 303, 304, 305 | F10 – F19 |
| **Schizophrenia spectrum disorders** | 295, 297-299 | 295, 297-298 | F20-F29 |
| **Bipolar disorders** | 296 [ excluding 295, 296.2] | 296 [excluding 295, 296B] | F30-F31[excluding F20] |

*NOTE.* † ICD Codes from the NPR.

**APPENDIX C. DESCRIPTIVE TABLE:** Descriptive characteristics of the cohort according to the following measures of crime: Type of crime, Number of convictions and Length of the sentences

| | | Type of crime † ‡ | | Number of Convictions§ | | | | | | Length of sentence (months) § | | | |
|---|---|---|---|---|---|---|---|---|---|---|---|---|---|
| **Individuals study cohort** | **Overall N (%)** | **Non-violent crime N (%)** | **Violent Crime N (%)** | **0 (%)** | **1 N (%)** | **2 -3 N (%)** | **4- 9 N (%)** | **10-19 N (%)** | **>20 N (%)** | **0 N (%)** | **1 to 11 N (%)** | **12 to 23 N (%)** | **>24 N (%)** |
| **Total** | 3,617,028 | 737,415 (20.39) | 118,155 (3.27) | 2,824,248 (78.08) | 454,983 (12.58) | 210,019 (5.81) | 95,871 (2.65) | 21,370 (0.59) | 10,537 (0.29) | 3,495,430 (96.61) | 94,887 (2.62) | 11,010 (0.30) | 16,701 (0.46) |
| Male | 1,847,582 (51.08) | 569,360 (77.21) | 106,545 (90.17) | 1,229,998 (43.55) | 326,613 (71.79) | 176,779 (84.17) | 85,467 (89.15) | 19,236 (90.01) | 9,489 (90.05) | 1,733,662 (49.61) | 88,212 (92.97) | 10,053 (91.31) | 15,655 (93.74) |
| Female | 1,769,446 (48.92) | 168,055 (22.79) | 11,610 (9.83) | 1,594,250 (56.45) | 128,370 (28.21) | 33,240 (15.83) | 10,404 (10.85) | 2,134 (9.99) | 1,048 (9.95) | 1,760,768 (50.39) | 6,675 (7.03) | 957 (8.69) | 1,046 (6.26) |
| **Dementia cases** | 56,590 (1.56) | 10,407 (1.41) | 1,556 (1.32) | 45,229 (1.60) | 6,821 (1.50) | 2,908 (1.38) | 1,247 (1.30) | 261 (1.22) | 124 (1.17) | 54,826 (1.57) | 1,466 (1.54) | 128 (1.16) | 170 (1.02) |
| Mean Age. diag. (95% CI) | 70.43 (70.38-70.49) | 69.16 (69.03-69.30) | 66.69 (66.32-67.07) | 70.79 (70.73-70.85) | 69.84 (69.68-70) | 68.67 (68.41-68.92) | 67.03 (66.62-67.44) | 64.41 (63.49-65.32) | 61.50 (60.25-62.74) | 70.54 (70.48-70.59) | 67.66 (67.30-68.02) | 65.75 (64.42-67.08) | 64.00 (62.76-65.24) |
| IRR 10,000 person years | 10.81 (10.72-10.90) | 11.19 (10.98-11.41) | 12.17 (11.58-12.79) | 10.67 (10.57-10.77) | 11.20 (10.94-11.47) | 11.46 (11.04-11.88) | 11.99 (11.34-12.67) | 12.96 (11.45-14.60) | 14.15 (11.81-16.79) | 10.76 (10.67-10.85) | 13.00 (12.35-13.68) | 11.08 (9.27-13.11) | 10.46 (8.96-12.11) |
| Male, N (%) | 27,978 (49.44) | 7,822 (75.16) | 1,392 (89.46) | 19,347 (42.78) | 4,822 (70.69) | 2,360 (81.16) | 1,099 (88.13) | 236 (90.2) | 114 (91.94) | 26,323 (48.01) | 1,372 (93.59) | 120 (93.65) | 163 (95.88) |
| Mean Age. diag. (95%CI) | 70.13 (70.05-70.21) | 69.07 (68.91-70.15) | 66.85 (65.45-67.25) | 70.66 (70.57-70.75) | 69.92 (69.73-70.11) | 68.69 (68.41-68.97) | 66.97 (66.54-67.4) | 64.41 (63.45-65.36) | 61.41 (60.12-62.7) | 70.31 (70.23-70.36) | 67.71 (67.34-68.09) | 66.06 (64.69-67.43) | 64.16 (62.89-65.43) |
| IRR 10,000 person years | 10.63 (10.50-10.75) | 10.98 (10.74-11.22) | 12.05 (11.42-12.69) | 10.39 (10.24-10.53) | 11.00 (10.69-11.31) | 11.07 (10.62-11.52) | 11.87 (11.18-12.59) | 13.00 (11.41-14.73) | 14.40 (11.91-17.21) | 10.52 (10.39-10.65) | 13.03 (12.35-13.73) | 11.31 (9.41-13.46) | 10.66 (9.10-12.38) |
| Female, N (%) | 28,612 (50.56) | 2,585 (24.84) | 164 (10.54) | 25,882 (57.22) | 1,999 (29.31) | 548 (18.84) | 148 (11.87) | 25 (9.58) | 10 (8.06) | 28,503 (51.99) | 94 (6.41) | <10 (6.25) | <10 (4.12) |

| | | | | | | | | | | | | | |
|---|---|---|---|---|---|---|---|---|---|---|---|---|---|
| Mean Age. diag. (95%CI) | 70.73 (70.65-70.81) | 69.17 (69.16-69.73) | 65.35 (64.17-66.54) | 70.88 (70.8-70.97) | 69.66 (69.34-69.97) | 68.58 (67.94-69.21) | 67.47 (66.22-68.71) | 64.44 (60.94-67.93) | 62.46 (57.08-67.84) | 70.74 (70.67-70.82) | 66.93 (65.47-68.39) | 61.06 (55.05-67.07) | 60.31 (53.53-67.09) |
| IRR 10,000 person years | 11.87 (10.87-11.13) | 11.89 (11.44-12.36) | 13.34 (11.40-15.49) | 10.90 (10.76-11.03) | 11.72 (11.22-12.24) | 13.51 (12.41-14.67) | 12.94 (10.96-15.14) | 12.61 (8.29-18.22) | 11.86 (5.94-20.80) | 11.00 (10.87-11.13) | 12.62 (10.24-15.35) | 8.47 (3.87-15.77) | 7.27 (3.12-14.07) |
| **MCI cases ¶** | 23.394 (0.65) | 4,896 (0.66) | 664 (0.56) | 18,005 (0.64) | 3,169 (0.70) | 1,434 (0.68) | 606 (0.63) | 122 (0.57) | 58 (0.55) | 22,647 (0.65) | 591 (0.62) | 65 (0.59) | 91 (0.55) |
| Mean Age. diag. (95%CI) | 67.83 (67.74-67.93) | 66.29 (66.07-66.50) | 64.52 (63.92-65.12) | 68.35 (68.24-68.46) | 67.03 (66.76-67.29) | 65.46 (65.06-65.86) | 63.83 (63.23-64.44) | 63.71 (62.26-65.16) | 61.72 (59.93-63.51) | 67.94 (67.85-68.05) | 64.65 (64.04-65.26) | 63.26 (61.24-65.28) | 63.08 (61.57-64.6) |
| IRR 10,000 person years | 4.46 (4.40-4.52) | 5.25 (5.11-5.40) | 5.18 (4.80-5.58) | 4.24 (4.18-4.30) | 5.19 (5.02-5.38) | 5.64 (5.35-5.94) | 5.36 (5.36-6.29) | 8.22 (7.03-9.54) | 6.60 (5.04-8.45) | 4.44 (4.38-4.49) | 5.2 (4.82-5.66) | 5.62 (4.36-7.10) | 5.59 (4.52-6.82) |
| Male, N (%) | 11,793 (50.41) | 3,606 (73.65) | 584 (87.95) | 7,780 (43.21) | 2,192 (69.17) | 1,159 (80.82) | 501 (82.67) | 108 (88.52) | 53 (91.38) | 11,091 (48.97) | 554 (93.74) | 59 (90.77) | 89 (97.80) |
| Mean Age. diag. (95%CI) | 67.80 (67.66-67.93) | 66.28 (66.03-66.53) | 64.59 (63.95-65.23) | 68.66 (68.51-68.82) | 67.23 (66.92-67.55) | 65.51 (65.08-65.95) | 63.71 (63.06-64.37) | 62.95 (61.48-64.42) | 61.82 (59.96-63.69) | 68.02 (67.88-68.15) | 64.68 (64.06-65.29) | 62.89 (60.74-65.05) | 63.10 (61.55-64.64) |
| IRR 10,000 person years | 4.44 (4.39-4.55) | 5.05 (4.89-5.22) | 5.04 (4.64-5.46) | 4.17 (4.07-4.26) | 4.99 (4.78-5.20) | 5.42 (5.12-5.74) | 5.40 (4.94-5.89) | 5.9 (4.88-7.12) | 6.67 (5.03-8.64) | 4.42 (4.34-4.51) | 5.25 (4.82-5.70) | 5.55 (4.25-7.09) | 5.81 (4.68-7.10) |
| Female, N (%) | 11,601 (49.59) | 1.290 (26,35) | 80 (12.05) | 10,225 (56.79) | 977 (30.83) | 275 (19.18) | 105 (17.33) | 14 (11.48) | 5 (8.62) | 11,556 (51.03) | 37 (6.26) | <10 (9.23) | <10 (2.2) |
| Mean Age. diag. (95%CI) | 67.87 (67.73-68.01) | 66.31 (65.88-66.74) | 64.01 (62.28-65.73) | 68.11 (67.96-68.25) | 66.56 (66.07-67.05) | 65.24 (64.29-66.18) | 64.39 (62.81-65.98) | 69.58 (64.52-74.65) | 60.65 (50.64-70.66) | 67.89 (67.75-68.03) | 64.23 (61.09-67.38) | 66.80 (59.86-73.75) | 62.33 (23.18-101.49) |
| IRR 10,000 person years | 4.45 (4.37-4.53) | 5.92 (5.61-6.25) | 6.48 (5.16-8.01) | 4.29 (4.21-4.38) | 5.72 (5.37-6.08) | 6.77 (6-7.60) | 9.17 (7.53-11.04) | 7.04 (3.96-11.40) | 5.9 (2.12-12.71) | 4.45 (4.37-4.53) | 4.96 (3.52-6.73) | 6.35 (2.52-12.87) | 0.7 (0.3-6.41) |

NOTE. Abbreviations: MCI: Mild Cognitive Impairment; IRR: Incidence Rate Ratio per 10.000 person-years †Type of crime is a time-varying exposure; ‡Type of crime: Individuals can contribute to both non-violent and violent types of crime; § Number of convictions and Length of the sentence are time-fixed exposures; ¶ For the MCI analyses the study cohort consisted of 3,616,338 individuals. There were 690 individuals delated from the main cohort due to a MCI diagnosis stablished before age 50 or because 0 days of follow up.

## APPENDIX D. SENSITIVITY ANALYSIS

**Table D.1.** Sensitivity analysis Dementia. Association between the length of sentence and number of convictions and Dementia as hazard ratios (HR) with 95% confidence intervals (CI).

| | | **Model 1** | **Model 2** | **Model 3** | **Model 4** | **Model 5** | **Model 6** | **Model 7** | **Model 8** | **Model 9** | **Model 10** |
|---|---|---|---|---|---|---|---|---|---|---|---|
| **Length of Sentence (months)** | 0 | Ref. | Ref | Ref. | Ref. | Ref. | Ref. | Ref. | Ref. | Ref. | Ref |
| | 1-11 | 2.49 (2.28-2.73) | 1.58 (1.44-1.73) | 2.39 (2.18-2.61) | 1.73 (1.58-1.90) | 2.31 (2.11-2.52) | 2.25 (2.06-2.46 | 2.42 (2.21-2.64) | 1.68 (1.53-1.84) | 2.42 (2.21-2.64) | 2.46 (2.34-2.60) |
| | 12-23 | 3.49 (2.68-4.56) | 2.10 (1.60-2.76) | 3.37 (2.58-4.40) | 2.25 (1.72 2.95) | 3.25 (2.49-4.24) | 2.94 (2.25-3.86) | 3.38 (2.59-4.41) | 2.34 (1.79-3.06) | 3.31 (2.54-4.33) | 3.39 (2.92-3.93) |
| | ≥ 24 | 1.11 (0.74-1.66) | 0.67 (0.45-1.02) | 1.10 (0.73-1.63) | 0.77 (0.52-1.15) | 1.04 (0.69-1.55) | 0.99 (0.66-1.48) | 1.06 (0.71-1.58) | 0.77 (0.51-1.15) | 1.02 (0.69-1.53) | 1.11 (0.85 -1.45) |
| **Number of convictions** | 0 | Ref. | Ref | Ref. | Ref. | Ref. | Ref. | Ref. | Ref. | Ref. | Ref |
| | 1 | 1.62 (1.56 1.69) | 1.33 (1.28-1.39) | 1.56 (1.50-1.62) | 1.43 (1.38-1.49) | 1.55 (1.49-1.61) | 1.54 (1.48-1.61) | 1.60 (1.53-1.66) | 1.47 (1.42-1.53) | 1.59 (1.53-1.66) | 1.60 (1.54-1.66) |
| | 2-3 | 2.33 (2.18-2.51) | 1.59 (1.48-1.71) | 2.24 (2.08-2.40) | 1.74 (1.62-1.88) | 2.16 (2.01-2.32) | 2.11 (1.96-2.27) | 2.26 (2.10-2.42) | 1.83 (1.70-1.97) | 2.22 (2.07-2.39) | 2.26 (2.11-2.43) |
| | 4-9 | 2.96 (2.59-3.38) | 1.70 (1.49-1.95) | 2.87 (2.51-3.28) | 1.88 (1.64-2.16) | 2.74 (2.40-3.13) | 2.54 (2.22-2.90) | 2.79 (2.45-3.19) | 1.97 (1.72-2.26) | 2.72 (2.38-3.11) | 2.82 (2.47-3.22) |
| | 10-19 | 5.54 (4.02-7.62) | 2.44 (1.74-3.42) | 5.28 (3.83-7.28) | 2.86 (2.07-3.96) | 5.05 (3.67-6.96) | 4.59 (3.33-6.32) | 5.23 (3.81-7.19) | 3.11 (2.25-4.28) | 4.72 (3.42-6.52) | 4.96 (3.57-6.90) |
| | ≥ 20 | 2.66 (1.27-5.55) | 1.39 (0.64-2.90) | 2.67 (1.27-5.61) | 1.72 (0.83-3.57) | 2.31 (1.10-4.84) | 2.63 (1.26-5.49) | 2.58 (1.24-5.40) | 1.42 (0.68-2.99) | 2.22 (1.04-4.72) | 2.83 (1.37-5.83) |

Note. Model 1: HR (95% CI) adjusted for sex and birth year. Model 2: Full Adjustment for all covariates. Model 3: HR (95% CI) adjusted for sex and birth year and educational attainment. Model 4: HR (95% CI) adjusted for sex and birth year and mental health disorders. Model 5: HR (95% CI) adjusted for sex and birth year and physical health disorders. Model 6: HR (95% CI) adjusted for sex and birth year and depression. Model 7: HR (95% CI) adjusted for sex and birth year and anxiety. Model 8: HR (95% CI) adjusted for sex and birth year and substance use disorder. Model 9: HR (95% CI) adjusted for sex and birth year and schizophrenia spectrum disorders. Model 10: HR (95% CI) adjusted for sex and birth year and bipolar disorder.

**Table D.2.** Sensitivity analysis Mild Cognitive Impairment. Association between the length of sentence and number of convictions and MCI as hazard ratios (HR) with 95% confidence intervals (CI).

| | | Model 1 | Model 2 | Model 3 | Model 4 | Model 5 | Model 6 | Model 7 | Model 8 | Model 9 | Model 10 |
|---|---|---|---|---|---|---|---|---|---|---|---|
| **Length sentence (months)** | 0 | Ref. | Ref | Ref. | Ref. | Ref. | Ref. | Ref. | Ref. | Ref. | Ref |
| | 1-11 | 1.91 (1.64-2.23) | 1.22 (1.05-1.43) | 1.91 (1.64-2.22) | 1.31 (1.12-1.53) | 1.74 (1.49-2.03) | 1.66 (1.43-1.94) 1.82 | 1.84 (1.58-2.14) | 1.27 (1.09-1.49) | 1.86 (1.60 2.17) | 1.81 (1.60-2.18) |
| | 12-23 | 2.36 (1.50-3.71) | 1.45 (0.93-2.27) | 2.38 (1.51-3.74) | 1.51 (0.96-2.36) | 2.15 (1.37-3.38) | 1.94 (1.23-3.05) | 2.26 (1.44-3.55) | 1.55 (0.99-2.44) | 2.25 (1.43-3.54) | 2.28 (1.47-3.56) |
| | ≥ 24 | 2.68 (1.74-4.14) | 1.70 (1.10-2.64) | 2.76 (1.79-4.26) | 1.82 (1.18-2.82) | 2.49 (1.61-3.83) | 2.30 (1.49-3.55) | 2.54 (1.65-3.92) | 1.79 (1.16-2.44) | 2.51 (1.63-3.88) | 2.64 (1.71-4.08) |
| **Number convictions** | 0 | Ref. | Ref | Ref. | Ref. | Ref. | Ref. | Ref. | Ref. | Ref. | Ref |
| | 1 | 1.65 (1.56-1.76) | 1.34 (1.26-1.42) | 1.61 (1.51-1.71) | 1.42 (1.34-1.52) | 1.56 (1.47-1.66) | 1.54 (1.45-1.63) | 1.62 (1.52-1.72) | 1.48 (1.39-1.58) | 1.62 (1.53-1.73) | 1.62 (1.53-1.73) |
| | 2-3 | 2.00 (1.79-2.24) | 1.35 (1.20-1.52) | 1.98 (1.77-2.22) | 1.45 (1.29-1.63) | 1.83 (1.63-2.05) | 1.73 (1.54-1.94) | 1.91 (1.71-2.15) | 1.54 (1.37-1.74) | 1.91 (1.71-2.15) | 1.93 (1.72-2.16) |
| | 4-9 | 2.70 (2.19-3.33) | 1.57 (1.28-1.96) | 2.75 (2.23-3.29) | 1.65 (1.33-2.05) | 2.47 (2.00-3.04) | 2.20 (1.78-2.72) | 2.52 (2.04-3.11) | 1.75 (1.41-2.17) | 2.50 (2.02-3.08) | 2.53 (2.05-3.12) |
| | 10-19 | 3.10 (1.75-5.49) | 1.47 (0.83-2.67) | 3.22 (1.82-5.70) | 1.62 (0.91-2.88) | 2.79 (1.58-4.94) | 2.45 (1.38-4.34) | 2.86 (1.62-5.06) | 1.70 (0.96-3.03) | 2.72 (1.53-4.81) | 2.78 (1.57-4.94) |
| | ≥ 20 | 2.45 (0.90-6.66) | 1.44 (0.52-3.86) | 2.64 (0.97-7.17) | 1.55 (0.57-4.23) | 2.19 (0.81-5.92) | 2.28 (0.84-6.21) | 2.34 (0.86-6.37) | 1.29 (0.48-3.52) | 2.20 (0.81-6.01) | 2.45 (0.90-6.66) |

Note. Model 1: HR (95% CI) adjusted for sex and birth year. Model 2: Full Adjustment for all covariates. Model 3: HR (95% CI) adjusted for sex and birth year and educational attainment. Model 4: HR (95% CI) adjusted for sex and birth year and mental health disorders. Model 5: HR (95% CI) adjusted for sex and birth year and physical health disorders. Model 6: HR (95% CI) adjusted for sex and birth year and depression. Model 7: HR (95% CI) adjusted for sex and birth year and anxiety. Model 8: HR (95% CI) adjusted for sex and birth year and substance use disorder. Model 9: HR (95% CI) adjusted for sex and birth year and schizophrenia spectrum disorders. Model 10: HR (95% CI) adjusted for sex and birth year and bipolar disorder.
